# Quantitative Visual Detection of Mercury Ions With Ratiometric Fluorescent Test Paper Sensor

**DOI:** 10.3389/fchem.2022.859379

**Published:** 2022-03-25

**Authors:** Mimi Fan, Zhihui Pan, Chunjuan Wang, Yang Guo, Jingran Sun, Mingzhu Liu, Bo Peng, Jin Wu, Yanjun Fang

**Affiliations:** ^1^ The Key Laboratory of Risk Assessment and Control Technology for Environment and Food Safety, Tianjin Institute of Environment and Operational Medicine, Tianjin, China; ^2^ College of Chemistry and Chemical Engineering, Northwest Normal University, Lanzhou, China

**Keywords:** gold nanoclusters, fluorescent probe, paper-based sensor, visual quantitative detection, mercury ion

## Abstract

A novel ratiometric fluorescence probe based on nitrogen-doped blue carbon dots (NCDs) and red gold nanoclusters (Au NCs) for mercuric ion (Hg2+) has been prepared and characterized. A user friendly fluorescent test paper based sensor combined with smartphone was fabricated for rapid visual and quantitative detection. Hg^2+^ can specifically bind to Au^+^ on the surface of Au NCs, leading to the quench of red fluorescence while the fluorescence intensity of the NCDs with blue fluorescence remained unchanged as a internal standard signal. The implement of paper-based sensor address some common drawback in analytical process such as the detection time, analysis cost. In a further demonstration, a homemade detection device with smartphone was used to qualify the Hg^2+^. After adding different concentration of Hg^2+^, red, purple, and blue colors were obtained on the detection zones of the fluorescent test paper. The Android App Color Grab was used to identify the red, green and blue (RGB) values of fluorescent color. The rapid visual and quantitative detection of Hg^2+^ was accomplished with the detection limit of 2.7 nM for fluorescence, 25 nM for smartphone and 32 nM for paper strip. The developed multi-mode detection platform was successfully applied to the detection of mercury ions in water samples with acceptable recoveries. The NCDs and Au NCs probe facilitate the one-site environmental monitoring for Hg^2+^ with “naked-eye” and smartphone.

## Introduction

The presence of heavy metal mercury ions in environmental pollutants and foodstuffs are of great concern (Irvine et al., 2017; Gao et al., 2021). Hg^2+^ is difficult to decomposed in the environment, can easily bioaccumulate in the human body and causing seriously harm to creatures and human beings (Liu et al., 2013; Ping et al., 2018). Owing to the strong binding capacity between Hg^2+^ and many proteins or enzymes *in vivo*, mercury accumulates in some organs of the body over time, which can lead to chronic poisoning (Onyido et al., 2004; Wang et al., 2020a). Therefore, it is necessary to develop a more rapid and simpler determination method for Hg^2+^ in environmental pollutants and food samples.

Conventional methods for Hg^2+^ determination include atomic absorption spectrometry (Erxleben and Ruzicka, 2005), inductively coupled plasma mass spectrometry (ICP-MS) (Serafimovski et al., 2008), fluorescence, colorimetric and electrochemical methods (Nazeeruddin et al., 2006; Fayazi et al., 2016), and surface-enhanced Raman scattering (Du et al., 2013). Although these instrumental methods have very good accuracy and accuracy in many detection scenes, most of them need expensive purchase and maintenance cost, time-consuming manipulation steps and special training for measurement accuracy, precision and sensitivity. Conventional fluorescence methods have the advantages of high sensitivity and selectivity, and easy implementation, which have been widely employed in Hg^2+^ assays (Chen et al., 2015). Furthermore, fluorescence methods based on nitrogen-doped carbon quantum dots (Fu et al., 2022), boron and nitrogen co-doped graphene quantum dots (Liu et al., 2020), and water-soluble N-acetyl-l-cysteine-capped CdTe quantum dots (Rezaei et al., 2017) have been successfully applied for mercury ion detection.

The single-emission fluorescence detection of Hg^2+^ has inevitable limitations with respect to sensitivity, probe concentration, instrument factors or matrix effects in complex samples (Agarwalla et al., 2016; Zang et al., 2016). To minimize these limitations, ratio fluorescence analysis has been developed in recent years. By monitoring the change in the fluorescence intensity ratio of two or more fluorescent materials at different emission wavelengths, the method is more suitable for tracking analytical targets as many background interferences can be corrected (Sun et al., 2013; Yao et al., 2013). As noble photoluminescent materials, carbon quantum dots (CQDs) and gold nanoclusters (Au NCs) have excellent characteristics over traditional fluorescent dyes, such as favorable water solubility and biocompatibility, good optical features. These properties have proven to be conducive to the construction of ration fluorescence sensors based on CQDs and Au NCs (Yan et al., 2016; Wang et al., 2018).

Recently, blood analysis, pesticides, environmental hazard monitoring, and heavy metal ion determination have already been carried out using paper sensors (Zhou et al., 2016; Wang et al., 2018) owing to their low cost, portability, and ease of operation. However, most of these detection with fluorescent strips only provide qualitative data. The red, green, and blue (RGB) values of the ratiometric fluorescent paper strips can be extracted using a smartphone colorimetry for quantitative analysis of anions and cations (Zhang et al., 2019; Wang et al., 2020b). The utilization of paper sensors enables sensitive, visible, low-cost, and rapid detection of Hg^2+^ in water. By developing various specific applications, the enhanced functions of smartphones can be easily utilized (Peng et al., 2020). Hence, a fluorescent strip integrated with a visual analysis system on a smartphone will present great application prospects in the field of quantitative analysis.

In this study, we have developed an effective ratio fluorescence analysis method for the detection of mercury ions in water samples. The method is portable, rapid and quantitative by using a fluorescent paper-based sensor and smartphone. The ratio fluorescence probes were composed of nitrogen-doped blue carbon dots (NCDs) and red fluorescent Au NCs. Due to the interaction between mercury and gold ions in a closed shell layer (Hg^2+^ (4f^14^5d^10^)-Au+ (4f^14^5d^10^)), Hg^2+^ can specifically bind to Au^+^ on the surface of Au NCs (Xie et al., 2010; Chang and Chiang, 2013), which results in the destruction of the red Au NCs and the gradual quenching of their fluorescence intensity. As an internal standard signal, the blue fluorescence of NCDs remain constant owing to their outstanding photostability and chemical inertness. Therefore, the fluorescent color shift from red to pink, pink to purple, and purple to blue can be easily differentiated by visual detection, as well as picked up using a smartphone under UV light for quantitative analysis. The fluorescent paper-based sensor combined with a smartphone colorimetric kit make the detection of Hg^2+^ simpler, convenient, cost-effective.

## Materials and Methods

### Reagents and Instruments

All the reagents were bought from Aladdin and the reagents were of analytical grade. Ultrapure water (18.2 MΩ cm) was obtained using the Millipore water purification system (Millipore, MA, United States).

Fluorescence data were recorded on a FluoroMax-4 Compact Spectrofluorometric (HORIBA, United States). Transmission electron microscopy (TEM) images were recorded using a JEOL 2020 transmission electron microscope. The UV-visible (UV-vis) absorption was obtained using T6 New Century spectrometer. Fourier transform infrared (FT-IR) spectroscopy measurements were recorded on a UERTEX-70 FT-IR spectrometer (Thermo-Fisher, United States). X-ray photoelectron spectroscopy (XPS) analysis was performed using an EscaLab 250xi spectrometer. Powder patterns of the samples were collected using a MiniFlex600 X-ray powder diffractometer. The RGB color intensity of the fluorescence photographs were captured using a smartphone under a WFH-204B portable UV lamp (365 nm).

### Preparation of NCDs and Au NCs

All glassware was washed with aqua regia (V_HCl_: V_HNO3_ = 3:1) before the start of each experiment. The NCDs and Au NCs were prepared according to a literature procedure (Xie et al., 2018) and (Xie et al., 2009), respectively.

### Fluorescence Detection of Hg^2+^

By introducing various Hg^2+^ concentrations (0.05–2.5 µM) into the above ratio fluorescent probe solution, the sensitivity of the response to Hg^2+^ was assessed. Under 400 nm excitation, the 410–780 nm fluorescence spectrum was recorded. Fluorescent probe solutions were formulated by mixing NCDs and Au NCs in 2 ml phosphate buffered saline (PBS) buffer (10 mM, pH = 7.4) at a fluorescence intensity ratio of 1:1. In a homemade closed box, color changes were observed under a 365 nm UV lamp. A variety of common metal cations, including Na^+^, K^+^, Ca^2+^, Ni^2+^, Co^2+^, Cd^2+^, Ba^2+^, Fe^3+^, Zn^2+^, Cr^3+^, Al^3+^, Ag^+^, Mg^2+^, Pb^2+^, and Cu^2+^ were selected to investigate their selectivity and anti-interference ability.

### Smartphone Colorimetric Detection of Hg^2+^

Different concentrations of Hg^2+^ were added into the 1:1 fluorescent probe solution, and under a 365 nm UV lamp, the corresponding fluorescent color images were captured using a homemade smartphone-based colorimetric device. The RGB values of the generated fluorescence color information were obtained using the free Android application Color Grab [loomatix (2021) Color Gr, 2021].

### Paper-Based Fluorescent Sensor for the Detection of Hg^2+^

After the fluorescent paper sensor was developed according to the literature (Xie et al., 2019), the adjusted ratio fluorescent probe solution was added to the hydrophilic area to make it appear uniform and stable on the paper-based sensor. Different concentrations of the mercury ion solution were added to the hydrophilic area of the paper sensor. The RGB values were captured and recognized by the smartphone on Color Grab. The quantitative relationship between the color intensity and concentration was established for quantitative detection.

### Analysis of Hg^2+^with Fluorescent Probe

Tap water and Yellow River water (Lanzhou, China) were used for the recovery experiments in this study. All Yellow River water and tap water samples were carried out on the same day of collection. All water samples were filtered twice with a 0.22 μm microporous membrane before used for experiments. Various concentrations of Hg^2+^ were added to the water samples, while the probe solution was used for detection. Three independent experiments were performed to determine.

## Results and Discussion

### The Design of the Ratio Fluorescent Probe

**SCHEME 1:**
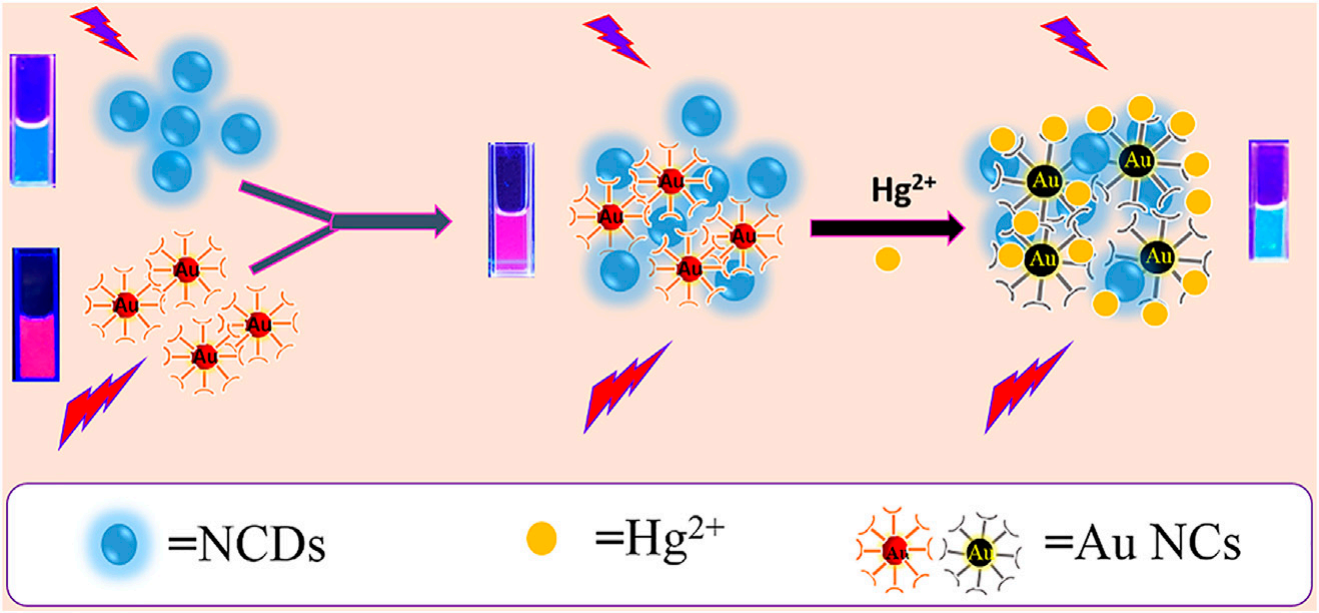
Schematic diagram of the formation principle and visualization of the ratio fluorescent probe for the detection of Hg2+.

In the current design strategy, the mixing ratio of NCDs and Au NCs was adjusted to realize the visual mercury ion detection. The reaction mechanism is shown in Scheme 1. The NCDs play a background reference role in the sensing system, while the Au NCs were quenched at the Hg^2+^ reaction site. The working principle presents a special high affinity between Hg^2+^ and Au^+^ interactions; hence, the red fluorescence of Au NCs is quenched by Hg^2+^ (Yan et al., 2016; Sahu et al., 2020), while the blue fluorescence of NCDs is stable against Hg^2+^, leading to distinct ratiometric fluorescence changes when exposed to Hg^2+^(Scheme 1).

### Preparation of the Paper-Based Fluorescent Sensor

**FIGURE 1:**
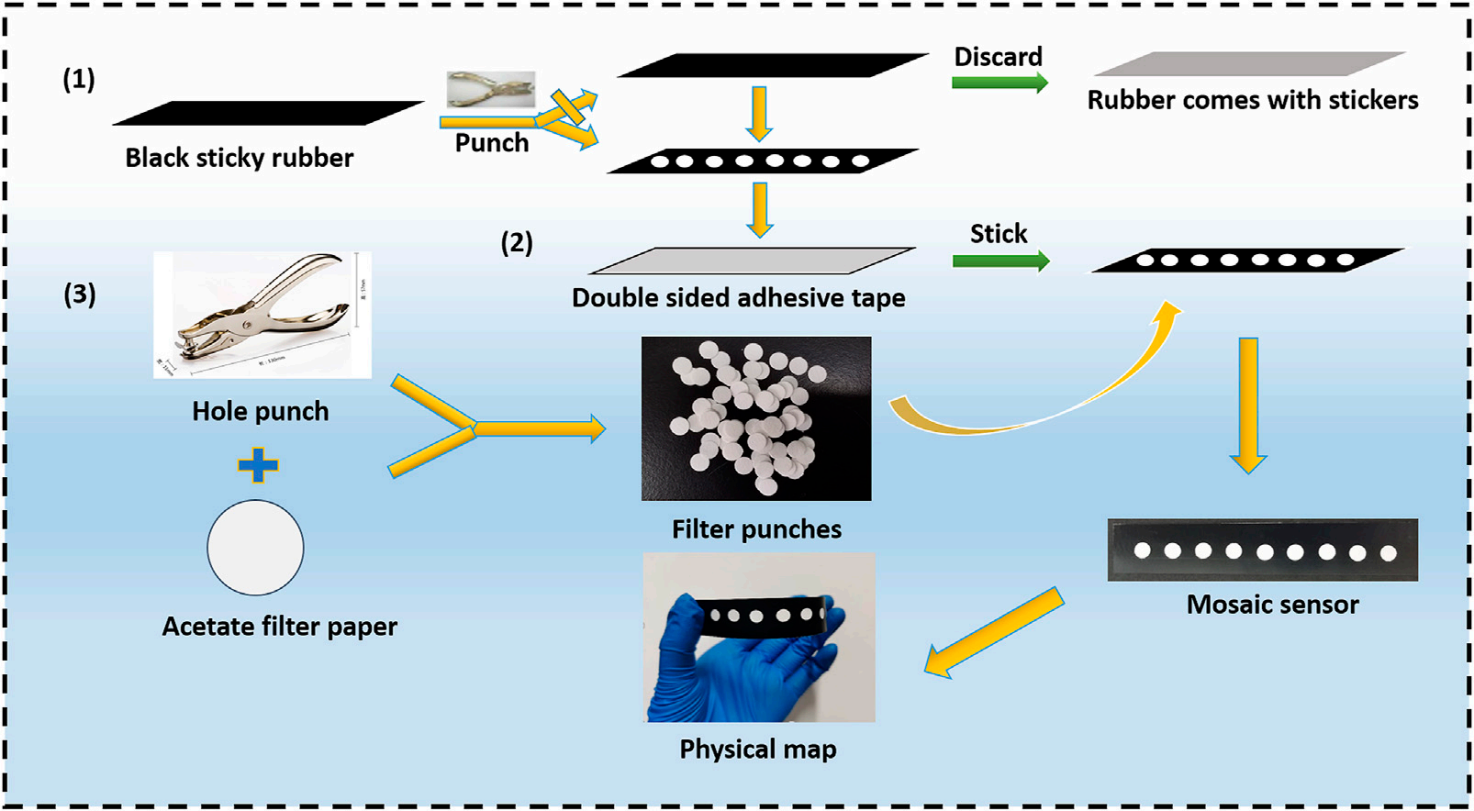
Schematic diagram of the paper-based sensor design.

For rapid mercury ion detection, the paper-based sensor was designed according to reference (Xie et al., 2019). The preparation process is as follows: by using a hand-held puncher, 6 mm holes were punched into the fluorescent acetate filter paper to form hydrophilic reaction zones. Subsequently, 6 mm diameter holes were also punched into the sub-light black film paper, discarding the self-adhesive black paper and pasting together the two sub-optical black film paper sheets to form the hydrophobic barrier area. Finally, the filter paper containing the 6 mm diameter holes was pasted onto two sub-optical black film paper sheets to construct a fluorescent paper-based sensor with good affinity and water drainage. The construction process is illustrated in Figure 1.

### Characterization of NCDs and Au NCs

The properties of the NCDs and Au NCs were characterized using TEM, UV-vis absorption spectroscopy, FT-IR spectroscopy, X-ray diffraction (XRD), XPS, and fluorescence spectroscopy. The NCDs and Au NCs were approximately 3–5 nm in size (Supplementary Figure S1A,B), which proves that the synthesized nanomaterials have uniform particle size and dispersion distribution in aqueous solution. The characteristic peaks at wavenumbers 1171 cm^−1^, 1310 cm^−1^, and 1390 cm^−1^ were assigned to the stretching vibrations of the C–O–C, C–N, and C–C bonds. The peak at 1650 cm^−1^ was assigned to the stretching vibration of C=O, while 2950 cm^−1^ and 3410 cm^−1^ were assigned to the stretching vibrations of the C–H or N–H/O–H bonds (Supplementary Figure S1C). A broad peak at 2θ = 19.48° was observed owing to the disordered carbon atoms and graphite lattice spacing (Supplementary Figure S1D). In the excitation wavelength range of 290–400 nm, the fluorescence intensity of the NCDs (Supplementary Figure S2A) varied with different amplitudes and reached a maximum at 360 nm. Similarly, when the excitation wavelength was adjusted to 330–410 nm, the fluorescence intensity of the Au NCs showed the same trend and reached the maximum value of 350 nm (Supplementary Figure S2B). To balance the excitation wavelength between the NCDs and Au NCs, 400 nm was selected as the optimal excitation wavelength. This clearly shows that the NCDs contain C, H, N, and O elements. The appearance of peaks at 284.77, 399.87, and 531.29 eV are assigned to the characteristic peaks of C 1s, N 1s and O 1s, respectively, which correlate well with the above FT-IR results (Supplementary Figure S2C). The high-resolution C 1s spectrum consists of three peaks at 284.71, 286.03, and 287.62 eV, which belong to the C−C, C−O, and C=O/COOH bonds, respectively (Supplementary Figure S2D). The high-resolution spectrum of the carbon point N 1s can be divided into three peaks at 398.97, 399.87, and 401.46 eV, related to the C−N−C, N−H and N−C=C bonds (Supplementary Figure S2E). The high-resolution spectrum of O 1s contains two peaks at 531.28 and 533.01 eV (Supplementary Figure S2F), which are related to the C=O and C−OH/C−O−C bonds. XPS analysis showed that there were −OH, −COOH, and −NH_2_ functional groups on the surface of the carbon point. As shown in the fluorescence spectra of the NCDs, the Au NCs and ratio fluorescent probe solutions are represented by letters a, b, and c, respectively (Supplementary Figure S3A). The ratio fluorescent probe solutions disperse well in water and exhibits dual-emission bands at 464 and 662 nm under excitation at 400 nm. Supplementary Figure S3B shows the UV−vis spectra. The black line represents the UV−vis absorption spectrum of NCDs. An acromion at 278 nm may be due to the n–π* excitation of the C=O group on the surface of the NCDs. The red line represents the UV−vis absorption spectrum of the Au NCs. No absorption was observed in the UV region. The blue line represents the UV−vis absorption spectrum of the ratio fluorescent probe solutions. The fluorescence intensity ratios (I_464_/I_662_) of the probe solutions remained steady over 1 h, proving their excellent photostability (Supplementary Figure S3C). Besides, the probe solutions were processed by centrifugation and found to have no significant increase or decrease in fluorescence intensity, and its fluorescence color was found to remain unchanged when photographed with a 365 nm UV lamp, indicating the good stability and usefulness of these ratiometric probe solutions.

### Optimal Fluorescence Intensity Ratio

pH is above 8 or below 6, the fluorescence intensity ratio of the probe solutions start to increase (Supplementary Figure S3E). The fluorescence intensity of the probe solutions tends to be stable in the pH range 7–8. Therefore, 7.4 was selected as the optimal pH. Kinetic experiments showed that the probe solutions can respond to Hg^2+^ in less than 5 min (Supplementary Figure S3F). To further illustrate the effect of these two fluorescent materials, a Hg^2+^ solution with a concentration ranging from 0.05 to 2.5 µM was added to a solution of NCDs and Au NCs The signal responses were then recorded. With an increase in Hg^2+^ concentration, NCDs did not response toward Hg^2+^, and its fluorescence intensity and color remained unchanged (Supplementary Figure S4A), while Au NCs continuously responded toward Hg^2+^, and its fluorescence intensity gradually decreased (Supplementary Figure S4B). With regards to the fluorescence color, the red became darker and difficult to distinguish with the naked eye. When the fluorescence intensity ratios of the probe solutions (NCDs: Au NCs) were set to 1:1, 1:2, and 1:3, respectively (Supplementary Figures S4C–E), the response toward Hg^2+^ was examined, and color changes were recorded.

### Response of the Ratio Fluorescent Probe to Hg^2+^

**FIGURE 2:**
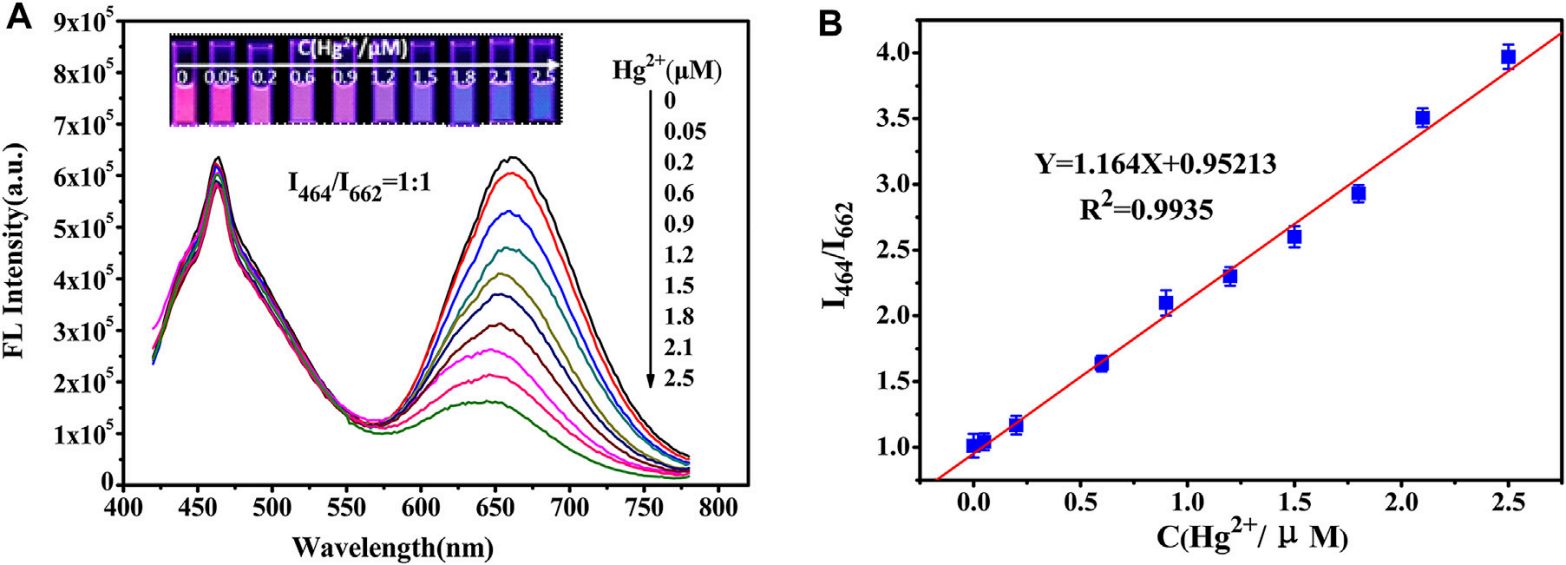
**(A)** Fluorescence spectra of the ratiometric fluorescent probes with the addition of Hg^2+^. The ratio of fluorescence intensity of NCDs to Au NCs is 1:1. The inset shows the corresponding fluorescence photographs taken under a 365 nm UV lamp. **(B)** Linear plot of the ratio of I_464_/I_662_ versus Hg^2+^ concentration.

A clear fluorescence color change from red to blue was observed (Supplementary Figure S4F) at a 1:1 ratio. Figure 2A illustrates the close relationship between the changes in fluorescence intensity and Hg^2+^ concentration. A good linear correlation (R^2^ = 0.9935) was obtained from the standard curve for Hg^2+^ concentrations ranging from 0.05 to 2.5 µM (Figure 2B). The limit of detection (LOD) for Hg^2+^ was 2.7 nM based on the 3δ/slope. The linear range and LOD results were compared with those obtained using other methods (Table 1).

**TABLE 1:** Comparison of the current probe with reported fluorescent probes.

Sensing systems	Year	Liner range (nM)	LOD (nM)	References
BCDs/RCDs	2018	0–320	0.14	Wang et al. (2018)
DTT/C-Au NCs	2018	50–1000	8.7	Liu et al. (2018)
CDs/CDs	2019	1–1000	0.22	Liu et al. (2019)
DNA/Au NPs	2020	50–2000	10	Mao et al. (2020)
CDs/BSA-Au NCs	2018	1–100	1.85	Yu et al. (2018)
CDs/Au NCs	2018	2–15	0.73	Xie et al. (2018)
C-dots/Au NCs	2016	0–500	28	Yan et al. (2016)
NCDs/Au NCs	2021	50–2500	2.7	This work

### Smartphone Colorimetric Detection of Hg^2+^

**FIGURE 3:**
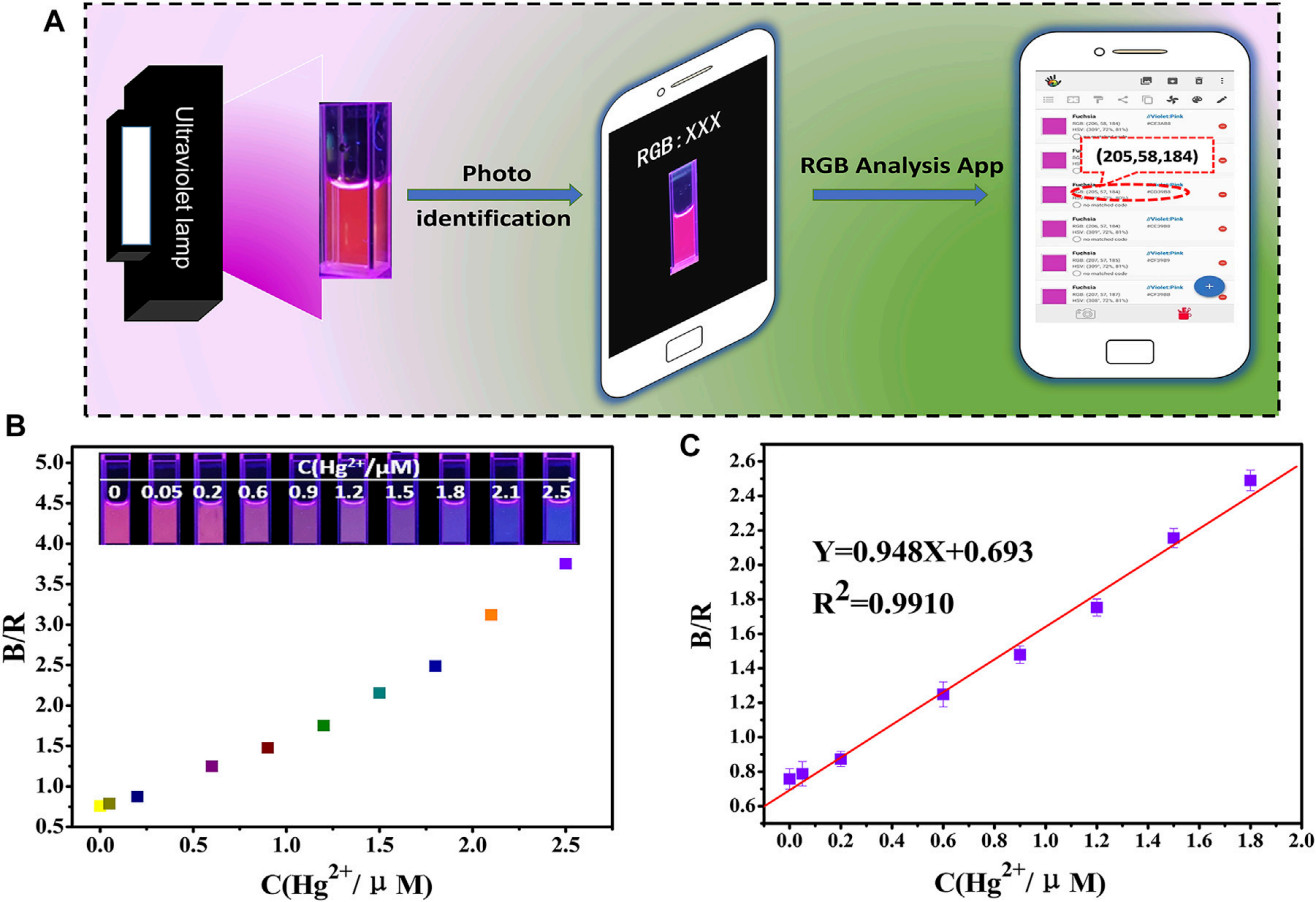
**(A)** Schematic drawing of the detection of Hg^2+^ using a smartphone; RGB analysis of the generated images via a color recognizer application; **(B,C)** detection of Hg^2+^ by the fluorescence probe solution combined with a smartphone.

As the Hg^2+^ concentration increase from 0.05 to 2.5 µM, fluorescence images show a shift from red to blue under 365 nm UV light. Figure 3A shows a schematic of Hg^2+^ detection using a smartphone. Coupled with a 365 nm UV light, the RGB values of the solution were detected and recorded immediately, as shown in Figure 3B.

Based on the analysis of the response results, the ratio of the blue and red channels (B/R) were selected to calculate the signal response values. The linear relationship between B/R and the Hg^2+^ concentration (0.05–1.8 µM) was obtained as shown in Figure 3C, with a linear equation of B/R = 0.948C + 0.693. Therefore, it seems that there is a good linear relationship between the signal response values and the Hg^2+^ concentration. The LOD was calculated to be 25 nM based on the 3δ/slope.

### Detecting Hg^2+^ With the Paper-Based Fluorescent Sensor

**FIGURE 4:**
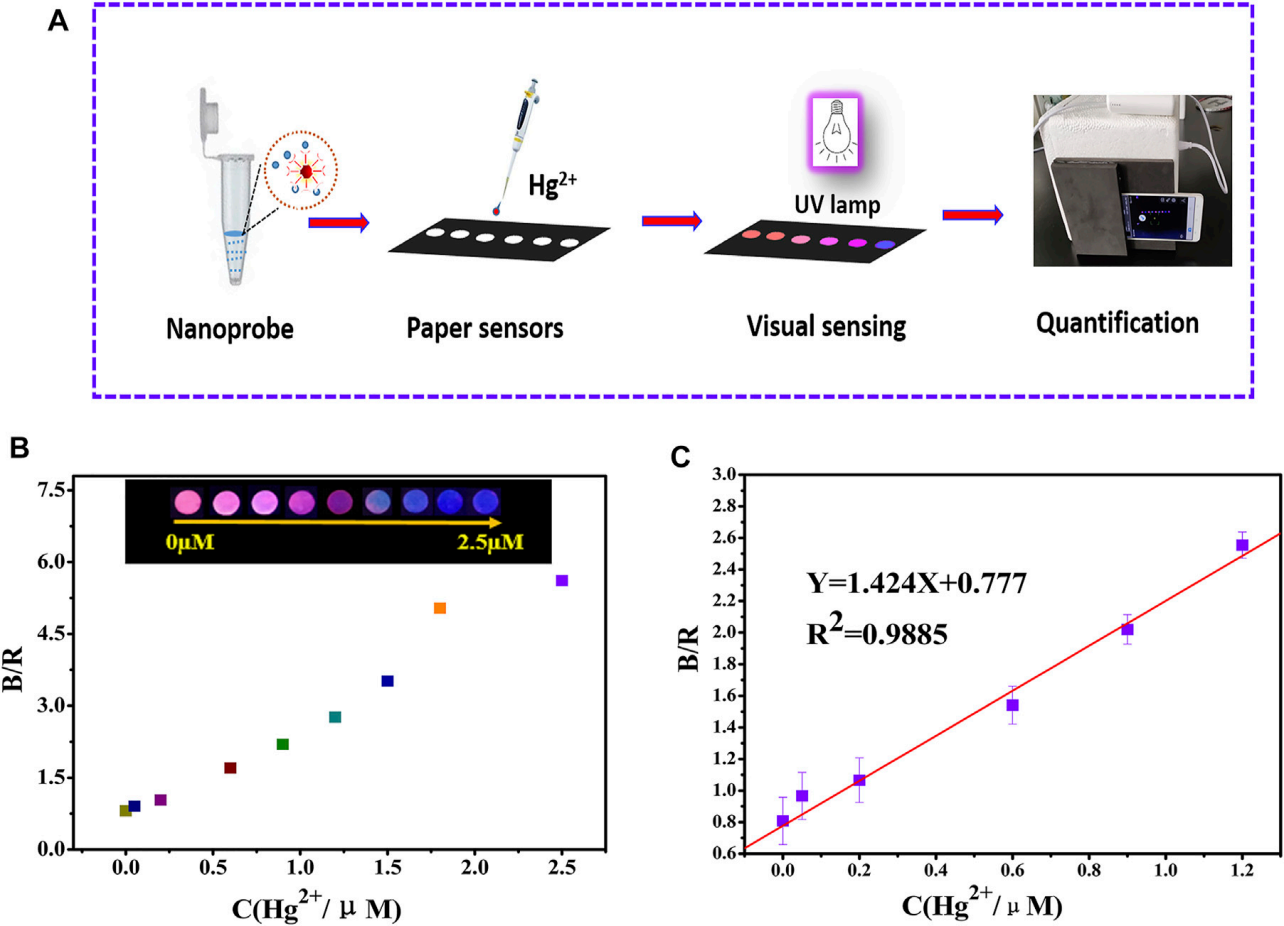
**(A)** The schematic diagram of visual and quantitative detection of mercury ions by the paper-based sensor platform; **(B)** smart phone recognition paper-based sensor detection of mercury ions and data **(C)**.

To facilitate the visualization of Hg^2+^, the probe solution was repeatedly and uniformly added dropwise to the paper-based sensor. Under a 365 nm UV lamp, the paper-based sensor exhibited a highly uniform red luminescence. Figure 4A shows a schematic diagram of mercury ion detection by the paper-based sensor. The paper color appears as a gradient of red to pink violet and finally to dark blue with an increase in Hg^2+^ concentration from 0.05 to 2.5 µM (Figure 4B).

The RGB values of the test paper were identified using the application Color Grab. The results are shown in Figure 4B, and good linearity was observed through the fitted linear equation (B/R = 1.424C + 0.777, R^2^ = 0.9885) when the Hg^2+^ concentration was in the range of 0.05–1.2 µM. The LOD was 32 nM based on the 3δ/slope (Figure 4C). As shown in Supplementary Figure S5C, the paper-based sensor was in the fluorescence color maintenance stage within 20 days at room temperature, and a distinct color variance still reemerged in the presence of 2.5 µM Hg^2+^.

In contrast, when different concentrations of mercury ion solutions were added to the paper-based sensor with the NCDs single probe solution, the blue fluorescence did not change under a 365 nm UV lamp (Supplementary Figure S5A), while the red fluorescence of Au NCs gradually weakened until it completely faded (Supplementary Figure S5B). It is difficult for the naked eye to separate color variations; however, the dual-emission ratio fluorescent paper sensor has a wide color range and is more suitable to observe for naked eye (Supplementary Figure S4F). These data suggest that the fluorescent paper chip sensor can simplify the detection process and achieve quantitative analysis of mercury ions using smartphones.

### Selectivity and Anti-interference Ability

**FIGURE 5:**
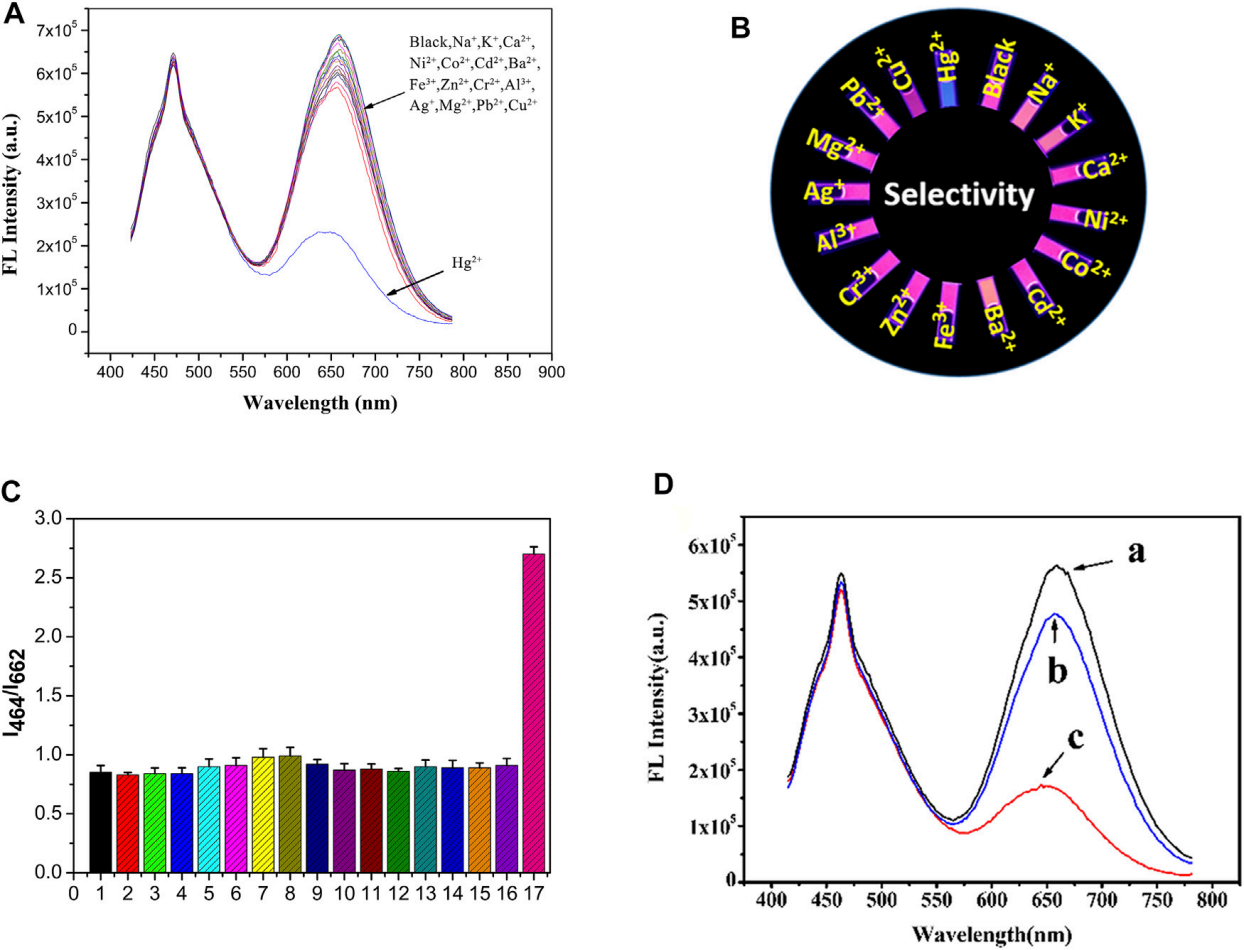
**(A)** Schematic diagram of the selective responses of the ratio fluorescent probe solutions to metal ions; **(B)** The corresponding fluorescence photos; **(C)** The histogram shows the selective responses of the fluorescence intensity (I464/I662) of the probe solutions to different metal cations, samples marked 1–17 represent 1. Blank, 2. Na^+^, 3. K^+^, 4. Ca^2+^, 5. Ni^2+^, 6. Co^2+^, 7. Cd^2+^, 8. Ba^2+^, 9. Fe^3+^, 10. Zn^2+^, 11. Cr^3+^, 12. Al^3+^, 13. Ag^+^, 14. Mg^2+^, 15. Pb^2+^, 16. Cu^2+^, 17. Hg^2+^; **(D)** The anti-interference performance of the ratio fluorescent probes; **(A)** without any addition of metal ions. **(B)** The addition of 25 μM of metal cations mentioned above together. **(C)** A subsequent addition of 2.5 μM Hg^2+^ in **(B)**.

As shown in Figures 5A,C, the fluorescence intensity at 662 nm increased up to 80% when the Hg^2+^ concentration was at 2.5 µM. The value of I_464_/I_662_ did not change significantly with the addition of 25 μM of other metal cations (Na^+^, K^+^, Ca^2+^, Ni^2+^, Co^2+^, Cd^2+^, Ba^2+^, Fe^3+^, Zn^2+^, Cr^3+^, Al^3+^, Ag^+^, Mg^2+^, Pb^2+^, and Cu^2+^). Therefore, the fluorescent probe solution has a high selectivity toward Hg^2+^ because of the specific binding between the mercury ions and the functional groups on the Au NCs.

With the addition of 1 μM of the metal cations, the I_464_/I_662_ ratio increased by approximately 15% (Figure 5D). However, this phenomenon was significantly different after the addition of mercury ions. The corresponding fluorescence images (Figure 5B) were also photographed using a 365 nm UV lamp. The data suggests that the ratio fluorescent probe solution exhibits excellent selectivity and anti-interference capability for Hg^2+^. Cu^2+^ ion shows slight suppression effect on BSA-AuNCs fluorescence mainly results from its low concentration in the selectivity and anti-interference experiment. The pre-separation method should be adopted if the matrixes contain large amount of Cu^2+^ ion higher than 10 times.

### Detection of Hg^2+^ in Real Samples

The ratio fluorescence probe solution was applied to determine the Hg^2+^ in tap water (from our laboratory) and Yellow River water samples (from An Ning, Lanzhou.). All the water samples were treated with ordinary qualitative filter paper and 0.22 μm Supor filters. Hg^2+^ at concentrations of 0.5, 1.2, 1.6, and 2.0 μM were spiked to test the rate of recovery. The results are shown in Table 2. The recovery rates obtained ranged from 90.5 to 107.6%. The results indicate that the designed probe solution has practical applicability for the detection of Hg^2+^ in water samples.

**TABLE 2:** The rate of recovery of Hg^2+^ in water samples (Using luminescence spectrometer).

Spiked Concentration (μM)	Tap water			Yellow River water		
	Found (μM, *n* = 3)	Recovery (%, *n* = 3)	RSD (%, *n* = 3)	Found (μM, *n* = 3)	Recovery (%, *n* = 3)	RSD (%, *n* = 3)
0.5	0.48	96.0	2.9	0.47	94.0	2.2
1.2	1.24	103.3	3.1	1.29	107.6	5.1
1.6	1.71	106.9	4.2	1.57	98.1	3.9
2.0	2.09	104.5	2.7	1.81	90.5	3.1

## Conclusion

In short, the designed dual-emission ratio fluorescent probe exhibits two characteristic emission peaks at 464 and 662 nm under 400 nm excitation by adjusting the fluorescence intensity ratio to 1:1. The fluorescence colorimetric signal (from red to blue) can be attributed to the variation in the fluorescence intensity ratio, which can be easily observed by the naked eye under an UV lamp. Compared to the individual probe, the fluorescence probe has good sensitivity and reliability. The ratio fluorescence probe can also be used for the fluorescence detection of Hg^2+^ in water samples. At the same time, using a smartphone to identify the RGB values of the probe solution can simplify the analysis equipment and achieve rapid colorimetric detection of mercury ions. The utilization of paper sensors with the assistance of a smartphone enables sensitive, visible, low-cost, and rapid detection of Hg^2+^ in aqueous medium.

## Data Availability

The original contributions presented in the study are included in the article/Supplementary Material, further inquiries can be directed to the corresponding authors.
