# Supplementary material for: Quantitative Visual Detection of Mercury Ions With Ratiometric Fluorescent Test Paper Sensor
Source: Front Chem. 2022 Mar 25;10:859379. doi: 10.3389/fchem.2022.859379 (PMC8990869; doi:10.3389/fchem.2022.859379)
Supplement: Supplementary file 1 [file DataSheet1.docx]

**Rapid visual and quantitative detection of mercury ions using a ratiometric fluorescence probe and paper-based sensor combined with smartphone**

Mimi Fan ^1, 2^, Zhaolin Gu ^3^, Yang Guo ^2^, Jin Wu ^1^, Chunjuan Wang ^2^, Zhixian Gao ^1^, Jialei Bai ^1^, Bo Peng ^2, *^, Yanjun Fang ^1,*^

^1^ Tianjin Institute of Environment and Operational Medicine, the Key Laboratory of Risk Assessment and Control Technology for Environment and Food Safety, Tianjin 300050, P. R. China

^2^ College of Chemistry and Chemical Engineering, Northwest Normal University, Lanzhou 730070, China

^3^ Tianjin University of Technology and Education, Tianjin 300222, P. R. China


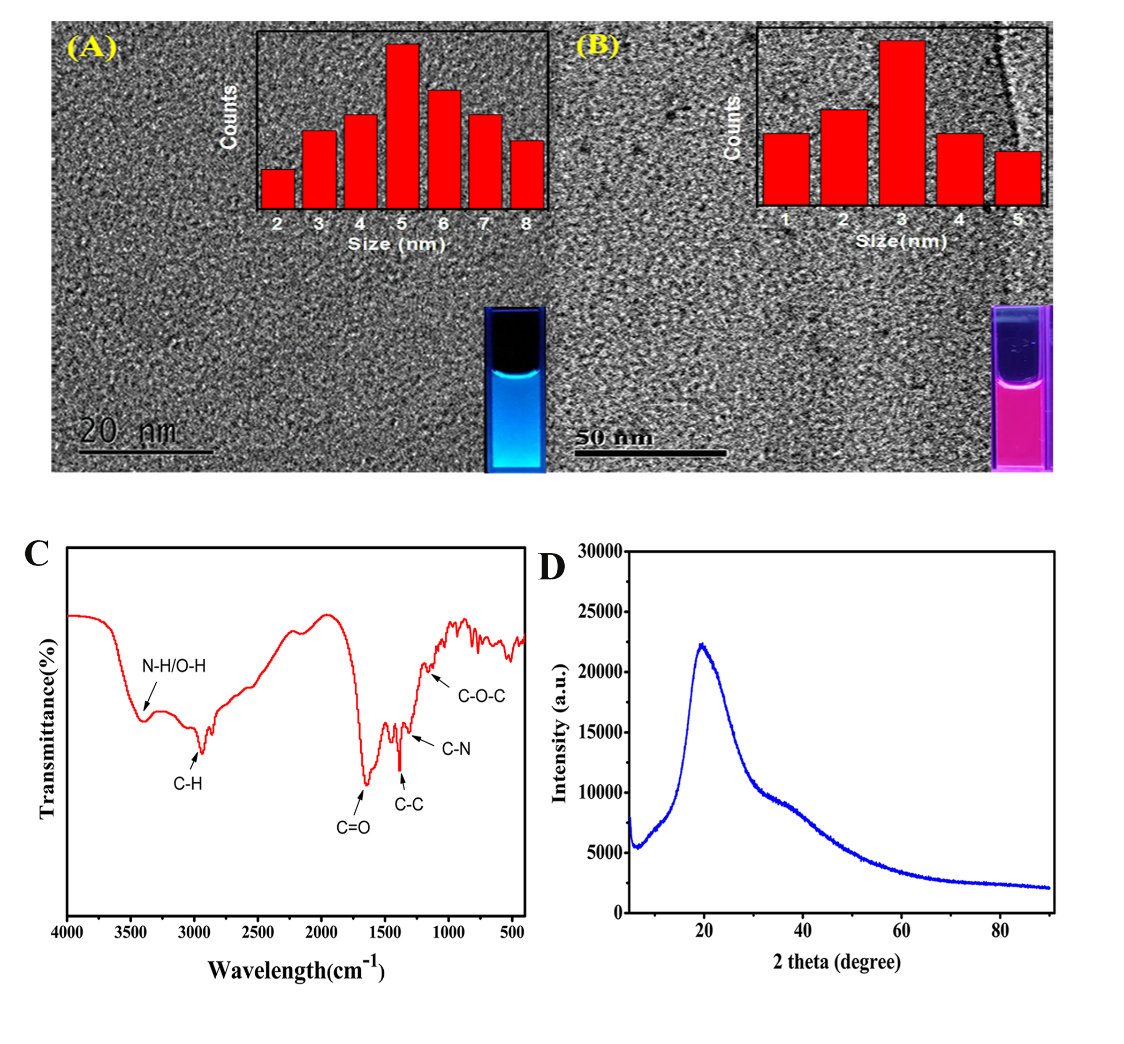


**Figure S1** The TEM images of nitrogen doped blue carbon dots (NCDs) (A), (B) gold nanoclusters (Au NCs), (C) FT-IR spectrum of nitrogen doped blue carbon dots (NCDs) and (D) XRD pattern of nitrogen doped blue carbon dots (NCDs).


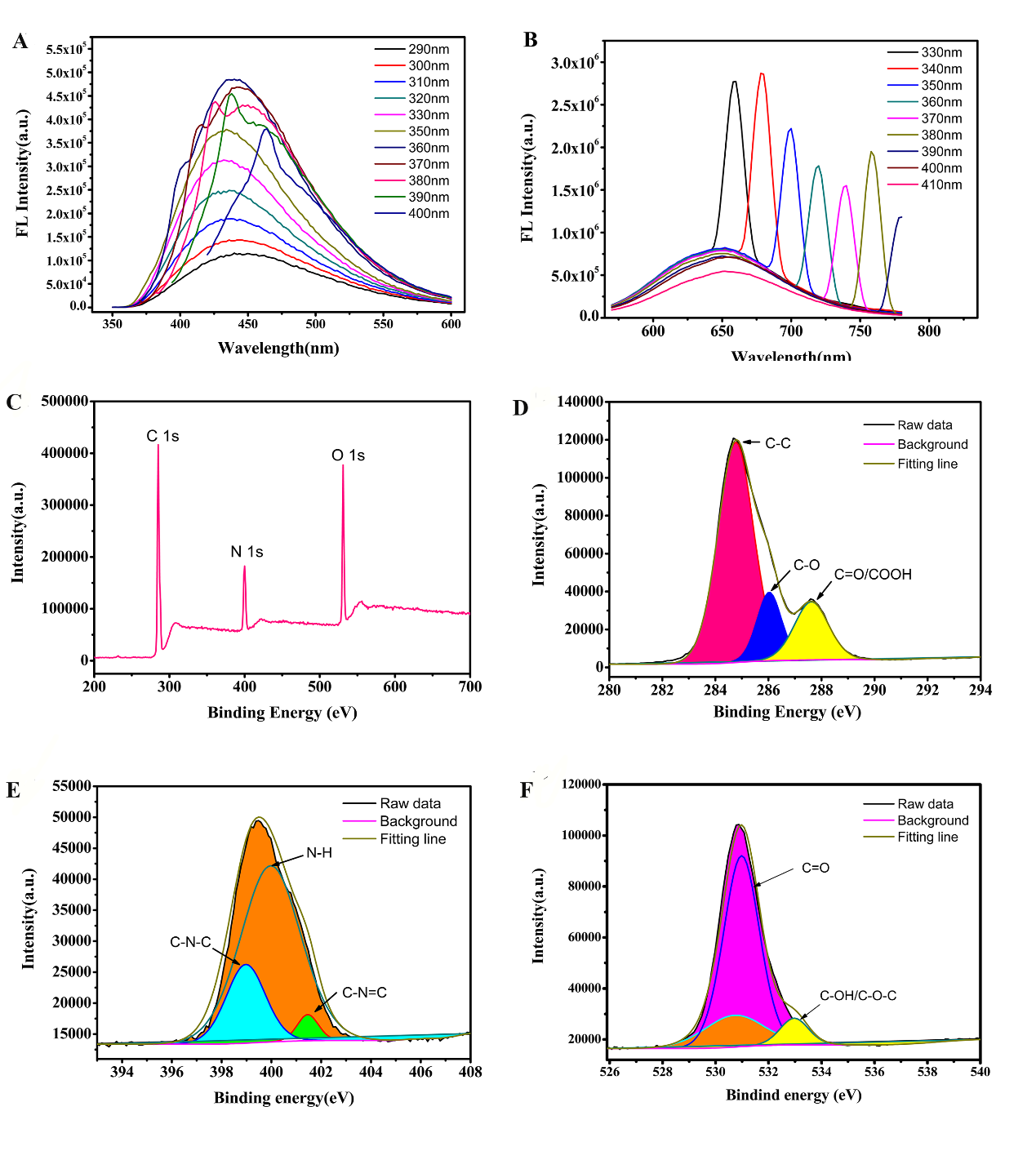


**Figure S2** (A) Fluorescence emission of nitrogen doped blue carbon dots (NCDs), (B) gold nanoclusters (Au NCs) at different excitation wavelengths, (C) XPS spectrum of nitrogen doped blue carbon dots (NCDs), (D) C 1s, (E) N 1s, (F) O 1s high-resolution XPS spectra.


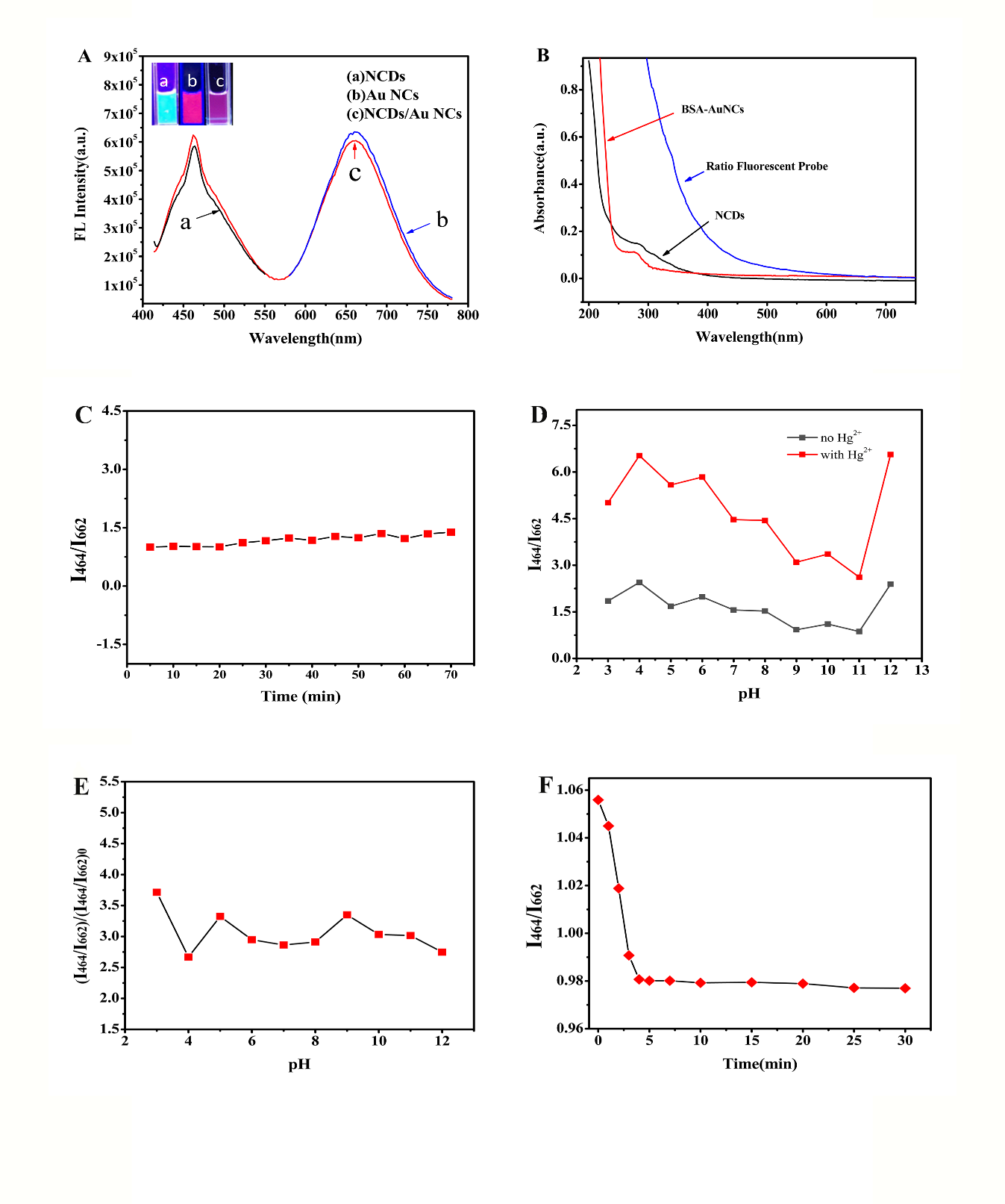


**Figure S3** (A) Fluorescence emission spectra (λex = 400 nm) of NCDs (a), Au NCs (b) and mixing NCDs and Au NCs (c), respectively, (B) UV-Vis absorption spectra of components in fluorescence system, (C) The light stability of the ratio fluorescent probe, (D) Effect of pH on fluorescence ratio probes (The red line represents the presence of Hg^2+^, the black line represents the absence of Hg^2+^)、(E) (I_464_/I_662_) is the fluorescence intensity ratio of adding 2.5 µM Hg^2+^，and (I_464_/I_662_)_0_ is of without Hg^2+^, (F) The temporal fluorescent response by the ratio fluorescence I_464_/I_662_ after the addition of 2.5 μM Hg^2+^.


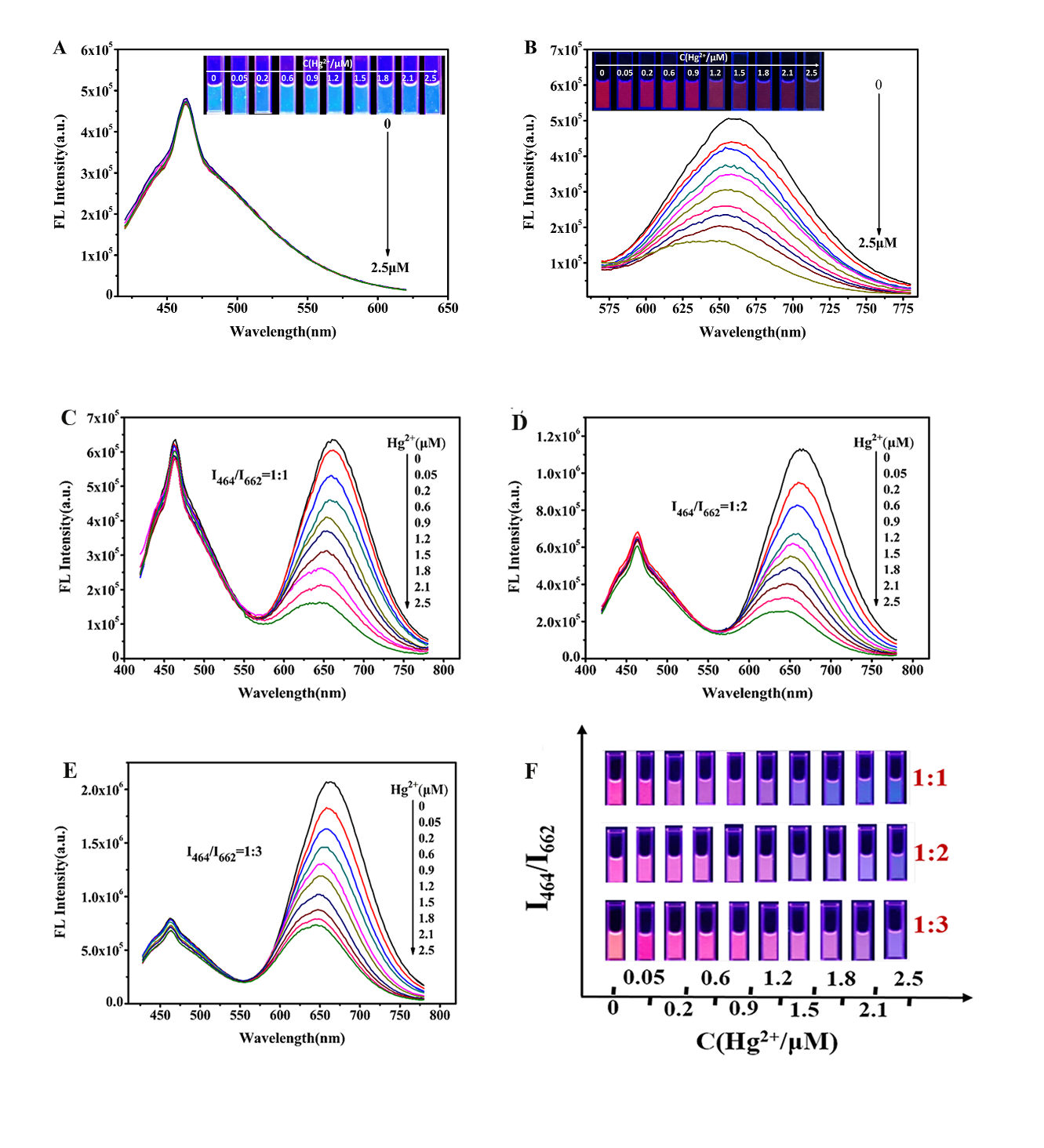


**Figure S4** The fluorescent emission spectra (λex= 400 nm) of (A) NCDs and (B) Au NCs with the addition of Hg^2+^, The inset photos show the corresponding color evolutions under a 365 nm UV lamp, (C) The fluorescent spectra of mixture of NCDs and Au NCs at ratios 1:1、(D)1:2、(E)1:3 with the addition of Hg^2+^; (F) The figure shows the corresponding fluorescence photo under 365 nm UV lamp.


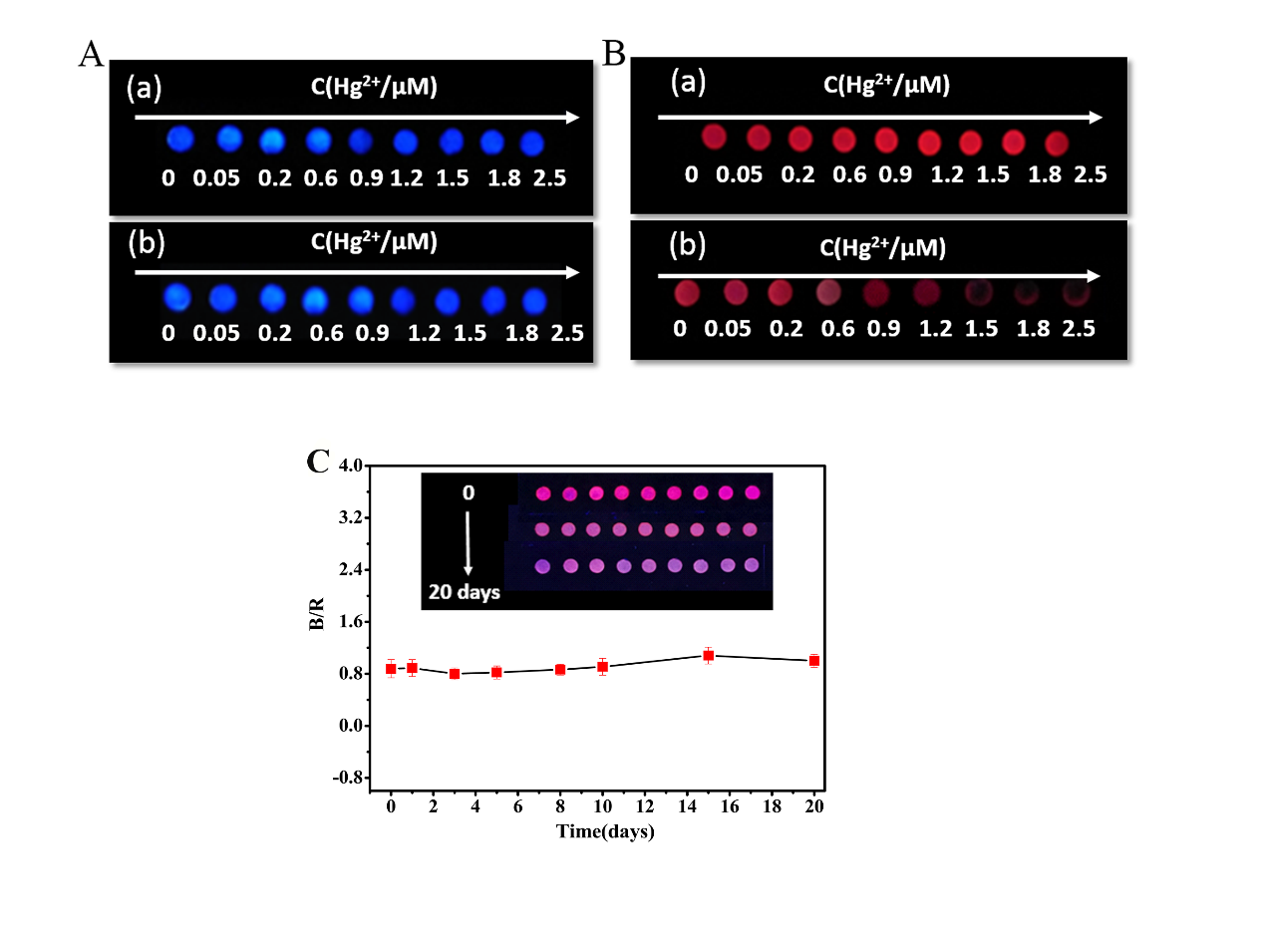


**Figure S5** (A) (B)The images of the paper sensors under a 365 nm UV lamp (a) no Hg^2+^ solution was added and (b) after addition of various concentrations of Hg^2+^ solution. The concentrations of Hg^2+^ from left to right were 0, 0.05, 0.2, 0.6, 0.9, 1.2, 1.5, 1.8 and 2.5 μM, (C) The stability of the fluorescent paper strip for 20 days under room temperature.
